# Articular cartilage and sternal fibrocartilage respond differently to extended microgravity

**DOI:** 10.1038/s41526-019-0063-6

**Published:** 2019-02-18

**Authors:** Jamie Fitzgerald, Jamie Endicott, Uwe Hansen, Cathleen Janowitz

**Affiliations:** 1 Bone and Joint Center, Department of Orthopedic Surgery, Henry Ford Hospital System, Detroit, MI 48202 USA; 2 Department of Orthopaedics and Rehabilitation, Oregon Health and Science University, Portland, OR 97239 USA; 3 Institute of Musculoskeletal Medicine (IMM), University Hospital of Münster, 48149 Münster, Germany

## Abstract

The effects of spaceflight on cartilaginous structure are largely unknown. To address this deficiency, articular cartilage (AC) and sternal cartilage (SC) from mice exposed to 30 days of microgravity on the BION-M1 craft were investigated for pathological changes. The flight AC showed some evidence of degradation at the tissue level with loss of proteoglycan staining and a reduction in mRNA expression of mechano-responsive and structural cartilage matrix proteins compared to non-flight controls. These data suggest that degradative changes are underway in the AC extracellular matrix exposed to microgravity. In contrast, there was no evidence of cartilage breakdown in SC flight samples and the gene expression profile was distinct from that of AC with a reduction in metalloproteinase gene transcription. Since the two cartilages respond differently to microgravity we propose that each is tuned to the biomechanical environments in which they are normally maintained. That is, the differences between magnitude of normal terrestrial loading and the unloading of microgravity dictates the tissue response. Weight-bearing articular cartilage, but not minimally loaded sternal fibrocartilage, is negatively affected by the unloading of microgravity. We speculate that the maintenance of physiological loading on AC during spaceflight will minimize AC damage.

## Introduction

The major load-bearing tissue within the joint is articular cartilage (AC). AC is exquisitely sensitive to changes in biomechanical loading (reviewed by Sanchez-Adams et al.^1^). Under normal conditions, chondrocytes synthesize a balance of extracellular matrix (ECM) components so that the ability to resist tensile and compressive forces is maintained. Deviation from the normal range of biomechanical forces,^2–5^ including complete unloading,^6–8^ tips the balance from maintenance to pathology typically leading to cartilage erosion and later osteoarthritis. While the effects of the biomechanical unloading environment of spaceflight on bone and skeletal muscle are well-studied,^9,10^ the effects on AC are largely unknown. The responses of AC to microgravity are important to define because it is clear from clinical studies that load-bearing AC is different from bone and skeletal tissue in that has a very poor capacity to restore damaged tissue.^11^ Consequently, microgravity-induced joint pathology could compromise flight crew mobility, interfere with mission activities, and accelerate short- and long-term joint degradation in flight personnel.

To investigate the effect of extended microgravity on AC, joint tissue from mice exposed to 30 days of Spaceflight in the BION-M1 flight was assessed for evidence of cartilage degradation. Our data suggest that spaceflight results in tissue degradation in load-bearing AC, but not in minimally loaded sternal fibrocartilage.

## Results

### Articular cartilage

**Fig. 1:**
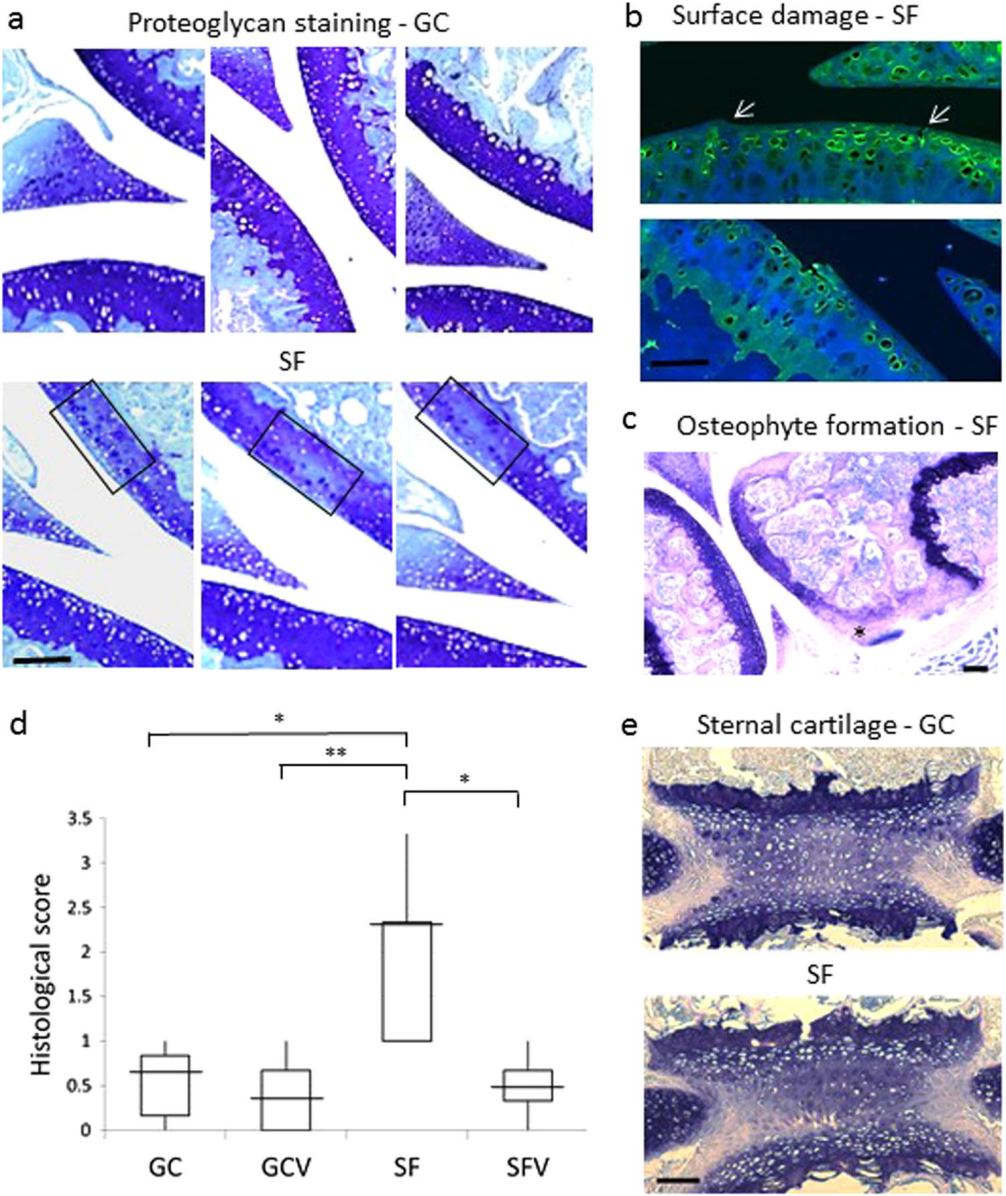
Histological analysis of spaceflight cartilage. **a** Proteoglycan analysis of articular cartilage. Sagittal sections of femoro-tibial joint were stained with toluidine blue for proteoglycan. The boxed region defines the area used to calculate the proportion of chondrocytes exhibiting pericellular proteoglycan in regions of reduced territorial proteoglycan. Scale bar is 100 μM. **b** Superficial zone damage. Sections were stained for collagen II. Several sections had surface irregularities. Note the uneven surface and fissure in the top panel (arrows) and damaged surface layer in the lower panel. Scale bar is 50 μM. **c** Presence of osteophytes. Representative image from a toluidine blue-stained SF sample showing evidence of an osteophyte (indicated by an asterisk) on the femoral condyle. Scale bar is 100 μM. **d** Histological scores for SF and non-flight controls (GC, GCV, and SFV). SF samples have a significantly higher histological score compared to ground and vivarium controls using the Kruskal–Wallis test (*P < 0.05, **P < 0.01). Median, maximum score, minimum score, and 25th and 75th percentile of total histology data are plotted for each experimental group. Scoring matrix is shown in Supplementary Table 1 and *P*-values for all pairwise combinations of experimental group histological scores shown in Supplementary Table 2. **e** Proteoglycan analysis of sternal cartilage. Representative sections of sternum from SF and GC mice cut in the coronal plane were stained for proteoglycan. The images show a single cartilaginous sternocostal synchondrosis flanked by the incoming ribs and the bony sternebrae above and below. Growth plate chondrocytes are located adjacent to the zones of calcified cartilage. Scale bar is 100 μM

**Fig. 2:**
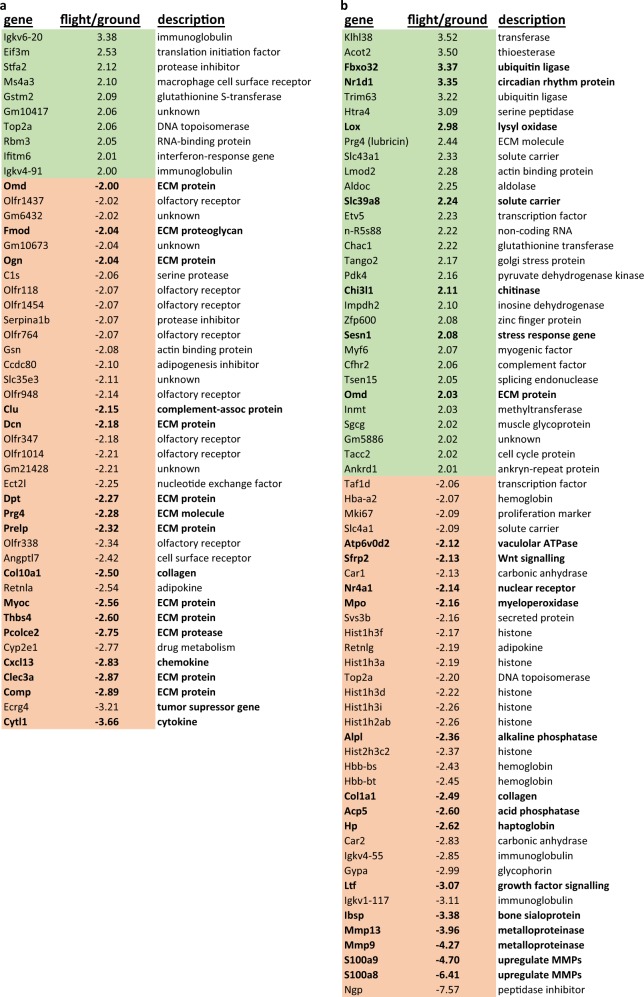
Cartilage RNA expression analysis. RNA isolated from articular cartilage **a** or sternal cartilage **b** were subjected to microarray analyses. Changes are expressed as fold change in flight compared to ground control samples. Only genes that are altered more than two-fold up in flight (shaded in green) and down in flight (shaded in orange) are listed. Structural cartilage extracellular matrix proteins or proteins associated with joint pathology are in bold

Spaceflight (SF) AC samples demonstrated less proteoglycan compared to AC ground controls (GC) (Fig. 1a). Decreased proteoglycan levels were generally restricted to the femoral condyle rather than the tibial plateau. Analysis of the boxed regions drawn around areas of reduced proteoglycan staining indicates approximately 35% of chondrocytes within the box stain for pericellular proteoglycan. Virtually no superficial zone chondrocytes have pericellular staining with the majority of toluidine blue-stained cells residing in the middle zone. Collagen II levels were similar between SF and control samples although three SF samples showed evidence of surface damage (Fig. 1b). In addition, clear evidence of osteophyte formation was present in three SF samples and in one GC and one SFV control sample (Fig. 1c). One SF femur sample was <2/3 the thickness of the average GC thickness. The SF mice had a significantly worse overall histological scores compared to all of the non-flight control groups, suggesting more overall cartilage degradation (Fig. 1d). Electron microscopy analysis of AC of elbows revealed no major differences in matrix density between SF and GC samples (Supplementary Fig. 1). Gene expression analysis showed several sustained changes in gene activation in SF compared to GC samples. Ten genes were upregulated and 37 genes downregulated greater than two-fold in flight compared to ground with a false discovery rate (FDR) of 0.05 (Fig. 2a). Seventeen of the altered genes are structural cartilage ECM proteins or proteins associated with joint pathology.

### Sternal cartilage

The availability of sternal tissue allowed us to compare the response of the two cartilages to microgravity. Representative images of the same cartilaginous sternocostal synchondrosis in the region between incoming ribs and ossified sternebrae are shown in Fig. 1e. This tissue is cartilaginous because it expresses the cartilage-specific gene *Col10a1* according to microarray gene expression data (not shown). In contrast to AC, there was no difference in extent and overall levels of proteoglycan staining between SF and GC samples in the sternal cartilage (SC), indicating no microgravity-induced proteoglycan decrease. Gene expression analyses demonstrated that 30 sternal genes were upregulated and 35 were downregulated in SF compared to GC (Fig. 2b).

## Discussion

The finding of reduced proteoglycan levels in AC due to microgravity is consistent with hindlimb unloading and limb immobilization studies in rodents, dogs, and rabbits, which consistently report unloading-specific AC atrophy in multiple experimental situations (reviewed in ref. ^8^). Our data suggest that despite partial proteoglycan reduction, the collagen II network remains intact. While surface damage and osteophytes were noted in several SF animals, there was no evidence of widespread AC fibrillation or fissure formation, and we conclude that exposure to microgravity results in moderate surface damage. Some chondrocytes were stained strongly for proteoglycan immediately surrounding the cell and may represent the production of new proteoglycan in the time elapsed (12–13 h) since returning to normal gravity although glycosaminoglycan synthesis assays are needed to confirm this.

Nine of the downregulated genes in spaceflight encode structural ECM components, including fibromodulin (*fmod*), osteoglycin (*Ogn*), osteomodulin (*Omd*), decorin (*Dcn*), dermatopontin (*Dpt*), PRELP (*Prelp*) collagen X (*Col10a1*), thrombospondin4 (*Tsp4*), and cartilage oligomeric matrix protein (*COMP*). Several other non-structural components with important cartilage ECM roles in development or osteoarthritis were also downregulated, including proteoglycan-related gene 4/lubricin (*Prg4*), procollagen C-endopeptidase enhancer 2 (*Pcolce2*), and Cytokine-like 1 (*Cytl1*). Cytl1 is an autocrine factor that regulates chondrogenesis in mesenchymal cells^12^ and is required for cartilage homeostasis.^13^ Expression of *Prg4* by superficial zone chondrocytes is acutely mechanosensitive; upregulation occurs with loading and associated with protection against osteoarthritis.^14–17^

The decrease in proteoglycan levels and downregulation of ECM molecules and genes that protect against osteoarthritic changes suggest that the early stages of cartilage breakdown are underway in the flight AC after 30 days of microgravity. However, since there is no evidence of significant collagen II degradation and the possible re-synthesis of proteoglycan in some chondrocytes, we suggest that cartilage recovery is possible and that 30 days of microgravity is insufficient for irreversible cartilage degradation.

In SC, there was no evidence of proteoglycan loss and a different suite of genes was altered in spaceflight. Of the downregulated genes, most notable were two prominent cartilage ECM-degrading enzymes: *Mmp13* and *Mmp9*. This finding together with the downregulation of S100a8 and S100a9, which are known to stimulate *Mmp* gene activation in cartilage,^18^ suggest that less ECM proteolysis occurs in SF samples compared to non-flight samples in SC tissue.

The expression of several genes was altered in both cartilage tissues, including *Prg4* and *Omd*, but opposite directions in the two cartilages. Osteomodulin regulates fibril diameter and is suggestive of new matrix synthesis in SC and reduced matrix production in AC.^19^ *Prg4* is chondroprotective and its upregulation in SC is further evidence that SC may be protected from degradation in microgravity.

Based on these differences in response to microgravity between the two cartilages, we suggest that the relative change in biomechanical environment determines the tissue response. During normal activities, AC is cyclically loaded with a significant fraction of body weight and then almost completely unloaded in microgravity. This change in loading triggers cartilage breakdown. In contrast, SC is loaded by cyclical lung expansion but does not experience the same magnitude of compressive loading as AC. Since the mice continue to breathe in microgravity and continuously load the tissue, the difference between mechanical loading in SF and controls is minimal, and cartilage breakdown is not initiated in SC.

Taken together, our findings suggest that maintenance of biomechanical loading during spaceflight will minimize AC destruction.

## Methods

### Animals

C57BL/6N male mice were flown for 30 days (477 Earth orbits) on the unmanned BION-M1 biosatellite between 19 April and 19 May 2013.^20^ Tissues were acquired from six male flight mice as part of NASA’s Biospecimen Sharing Program. The mice were specific pathogen-free and 19–20 weeks old at the time of launch and start of control experiments. In addition to the six flight mice (SF), there were eight “flight” vivarium male ground control mice (SFV), seven asynchronous ground control males (GC), and seven asynchronous vivarium ground control males (GCV). Tissues were harvested 12–13 h post-landing. Flight and animal habitat details for the BION-M1 mission have been reported by Andreev-Andrievskiy et al.^20^

IACUC approval was obtained from the MSU Institute of Mitoengineering and of the Biomedical Ethics Commission of IBMP and the study was conducted in compliance with the European Convention for the Protection of Vertebrate animals used for Experimental and Other Scientific purposes.

### Tissue analyses

Hindlimbs and elbow joints were dissected and right limbs placed in RNALater (Ambion) with left limbs placed in 10% neutral-buffered formalin (NBF). Sternae with ribs attached were cut mid-sternum and the posterior half containing xiphoid process and two sternocostal joints placed in RNALater and the anterior half placed in NBF for histology. AC processing, toluidine blue histology and immunohistochemistry, were performed as previously reported.^21^ AC sections were stained for Safranin-O (Saf-O) and counterstained with hematoxylin using a protocol from the University of Rochester, Center for Musculoskeletal Research (https://www.urmc.rochester.edu/musculoskeletal-research/core-services/histology/protocols.aspx). Cartilage thickness measurements were taken along the tibial and femoral articular surfaces of Saf-O positive AC at 30 different sites for each section in the same relative position throughout the joint and included calcified and non-calcified regions together. These within-animal measurements were used to derive an average thickness for each animal. The thickness variation within an experimental group was similar to the average variation within individual samples (not shown).

For transmission electron microscopy, cartilage samples from SF and GC elbows were dissected from bone and post-fixed in 0.5% (v/v) osmiumtetroxide and 1% (w/v) potassium hexacyanoferrate (III) in 0.1 M cacodylate buffer. After dehydration, specimens were incubated in propylenoxide and embedded in Epon. Ultrathin sections were cut, collected on copper grids, and negatively stained with 2% uranyl acetate. Electron micrographs were taken at 60 kV with a Phillips EM-410 electron microscope (Ditabis, Pforzheim, Germany).

### RNA analyses

RNALater-preserved AC from the femoral condyle and proximal tibia was dissected down to the calcified zone using an ophthalmic scalpel and tissue from SF and GC animals pooled separately. Half sternum pieces were dissected from ribs and SC dissected from bony segments and pooled. RNA was isolated using the MirVana RNA isolation kit (Life Technologies) and amplified by in vitro transcription with T7 RNA polymerase. Samples (100 ng) were then labeled using the Affymetrix GeneChip WT Plus protocol and hybridized to an Affymetrix Mouse Gene 1.0 ST GeneChip.

Analysis of microarray data was conducted using both Microarray Suite (MAS) version 5.0 and Robust Multi-Array Average (RMA).^22^ Differentially expressed genes were ≥−2 (decrease) or a fold change of ≥2 (increase) with an FDR of 0.05.

### Statistical analyses

For the AC histological scoring analysis, measurements were taken from seven GC, seven GCV, five SF, and six SFV samples where good joint histology in the correct plane was obtained. Histology scoring for SC was conducted on six SF, seven GC, eight SFV, and seven GCV animals. Each animal received a single histology score representing the sum of separate histological parameters shown in Supplementary Table 1 with higher scores representing more overall cartilage degradation. Statistical differences between experimental groups (GC, GCV, SF, SFV) for total histological scores from each animal were examined using the Kruskal–Wallis test for multiple independent samples (http://astatsa.com/KruskalWallisTest/).^23^ The omnibus *P*-value of 0.006 (d.f. = 3) rejects the null hypothesis that all groups have the same distribution. Post-hoc pairwise multiple testing by Dunn was used to determine which pairs are different.^24^ *P*-values were adjusted according to the family-wide error rate of Holm and then by the Benjamini–Hochberg method.^25^ *P-*values for all pairwise comparisons used to calculate overall Kruskal–Wallis *P*-values are shown in Supplementary Table 2. Median, maximum score, minimum score, and 25th and 75th percentile of total histology data plotted for each experimental group are shown in Fig. 1d.

### Reporting Summary

Further information on experimental design is available in the Nature Research Reporting Summary linked to this article.

## Supplementary information

Supplementary Material

Reporting Summary

## Data Availability

Microarray datasets generated and analyzed during the current study are available in the NASA GeneLab repository, https://genelab.nasa.gov/data/.

## Supplementary information

**Supplementary information** accompanies the paper on the *npj Microgravity* website (10.1038/s41526-019-0063-6).
