# Supplementary material for: Articular cartilage and sternal fibrocartilage respond differently to extended microgravity
Source: NPJ Microgravity. 2019 Feb 18;5:3. doi: 10.1038/s41526-019-0063-6 (PMC6379395; doi:10.1038/s41526-019-0063-6)
Supplement: Supplementary file 1 — Supplementary Material [file 41526_2019_63_MOESM1_ESM.pdf]

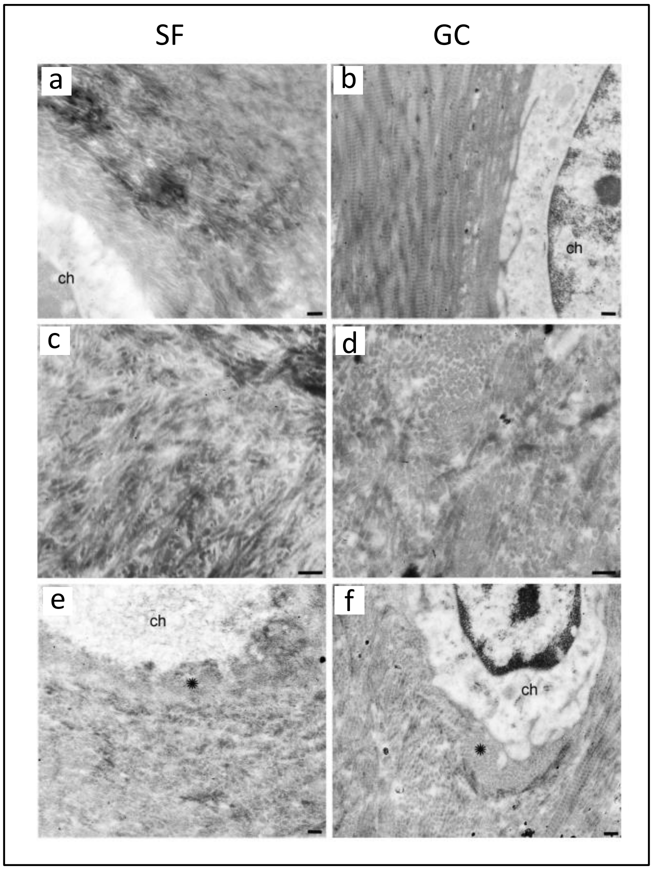

Supplementary Figure 1: **Ultrathin sections of cartilage of elbows.** Representative images of SF (a, c, e) and GC (b, d, f) samples. No major differences were found between the flight samples and ground control samples. However, the suprastructural organization of the pericellular matrix appears slightly altered in the flight samples (compare e and f, pericellular matrix is marked by stars). ch = chondrocyte. All bars are 200nm.

Matrix staining (toluidine blue)	Score
Normal	0
Slightly reduced	1
Markedly reduced	2
No staining	3
Surface regularity	
Smooth -100% to 75%	0
Moderate - 50% to 75%	1
Irregular - <50%	2
severely irregular	3
Cartilage thickness	
>2/3 depth relative to av. GC depth	0
1/2 to 2/3 depth relative to av. GC	1
<1/2 depth relative to av. GC	2
Osteophytes	
none	0
one	1
two or more	2

Supplementary Table 1: **Histological scoring matrix.** The matrix was used to determine the overall histological score shown in Fig. 1d. Sections were assessed using a histological scoring system based on our previously described scale[21]. The higher the total histology score (out of a maximum of 10), the more degraded the cartilage ECM. Sections were scored for proteoglycan loss and surface regularity by three independent and blinded observers and the score for the 'worst' section for each animal recorded.

GC-GCV	0.797
GC-SF	**0.015**
GC-SFV	0.909
GCV-SF	**0.007**
GCV-SFV	0.797
SF-SFV	**0.015**

Supplementary Table 2: **Kruskal-Wallis test *p*-values for all pairwise combinations of experimental groups for histological scores.** Values were adjusted using Dunn's post-hoc test and then further adjusted by the Benjamini-Hochberg method. Pairwise comparisons with a KW *P*-value <0.05 are highlighted in bold and indicated on the graph in Fig. 1d (*<0.05, **<0.01).
